# Injections of platelet‐rich plasma prepared by automatic blood cell separator combined with topical 5% minoxidil in the treatment of male androgenetic alopecia

**DOI:** 10.1111/srt.13315

**Published:** 2023-06-20

**Authors:** Wei Wei, Yuanjing Zhang, Binman Long, Yuanyuan Zhang, Chi Zhang, Siping Zhang

**Affiliations:** ^1^ Department of Dermatology Provincial Hospital Affiliated to Anhui Medical University Hefei China; ^2^ Department of Dermatology The First Affiliated Hospital of the University of Science and Technology of China Hefei China; ^3^ Department of Transfusion The First Affiliated Hospital of the University of Science and Technology of China Hefei China

**Keywords:** androgenetic alopecia, male pattern alopecia, platelet‐rich plasma, minoxidil

## Abstract

**Background:**

Platelet‐rich plasma (PRP) has been increasingly accepted as a potential therapy in the treatment of androgenetic alopecia (AGA), However, there remains a dearth of data on the effectiveness of PRP prepared by automatic blood cell separator with a combination of topical minoxidil for the treatment of AGA.

**Objective:**

To evaluate the efficacy and safety of PRP prepared by automatic blood cell separator combined with topical 5% minoxidil therapy in male AGA.

**Methods:**

Thirty male patients with mild/moderate AGA were enrolled in a randomized double‐blind controlled study. Patients were randomly divided into two treatment arms: (group A) PRP prepared by automatic blood cell separator combined with topical 5% minoxidil group; (group B) PRP prepared by automatic blood cell separator combined with a topical placebo group. Trichoscopic assessments regarding hair density/quantity and mean hair diameter were performed at baseline and follow‐up. Clinical efficacy of global photography and patient satisfaction were conducted to verify the therapeutic efficacy of the treatment, and the occurrence of adverse reactions was recorded.

**Results:**

We detected a significant increase in all patients in hair density and quantity after PRP treatment (*p* < 0.05), and there was no significant difference in mean hair diameter. Although hair density/quantity was more pronounced in group A than in group B, the difference between groups was not statistically significant (*p* > 0.05). In terms of clinical efficacy and patient satisfaction, group A was superior to group B, and no serious adverse reactions occurred.

**Conclusion:**

We hereby conclude that the injections of PRP prepared by an automated method are effective and safe in the treatment of mild‐to‐moderate male AGA patients, and its combination with topical 5% minoxidil therapy was superior to PRP monotherapy with better clinical efficacy and higher patient satisfaction.

## 1 INTRODUCTION

Androgenetic alopecia (AGA) is the most common type of non‐cicatricial alopecia in dermatology clinics. AGA has genetic susceptibility, which can occur in both men and women and has a certain influence on patients' mental health and social interaction, leading to the occurrence of anxiety, depression, and other adverse psychological conditions.^1^ At present, the pathogenesis of AGA remains unclear, androgen and heredity are the primary pathogenic factors. Current treatments for AGA include drug therapy, laser therapy, microneedle therapy, hair transplantation, and platelet‐rich plasma (PRP) therapy. The United States Food and Drug Administration‐approved treatments for AGA include oral finasteride and topical minoxidil.^2^ However, there are several adverse reactions of topical minoxidil, including dermatitis, scalp irritation, and prone to relapse after withdrawal, which requires long‐term use to maintain the efficacy. In addition, oral finasteride has side effects such as decreased libido, which limits clinical application. PRP with various growth factors is a concentrated platelet solution derived from the separation of plasma prepared from autologous venous blood, and it is enriched with various growth factors involved in the regulation of cell proliferation and differentiation and the induction of angiogenesis.^3^ PRP has been widely used with high safety and few adverse reactions.^4^ Studies have shown that PRP plays a positive role in the hair growth cycle and promotes hair regeneration, which can be used as a combined treatment for AGA.^5^, ^6^ Most previous studies have used traditional manual methods to prepare PRP, while blood samples prepared by automatic procedures have higher platelet concentrations than that by manual methods.^7^ The aim of this study was to evaluate the efficacy and safety of PRP prepared by automatic blood cell separator with a combination of topical 5% minoxidil therapy for male AGA.

## 2 MATERIALS AND METHODS

### 2.1 Study participants

From October 2021 to April 2022, 30 male patients with mild‐to‐moderate AGA were selected for a randomized double‐blind controlled study. Patients were randomly assigned to two treatment arms: PRP injection combined with topical minoxidil (Group A) and PRP injection combined with topical placebo (Group B). Patients were enrolled with blood routine examinations and filled in questionnaires, including age, duration of disease, previous history, treatment history, family history, etc. Patients were classified according to BASP classification (Basic and specific classification system)^8^ (Table 1). All patients ranged in age from 21 to 49 years old, with an average age of (30.41 ± 6.17) years old; The duration of disease ranged from 0.5 to 8 years. The average duration of the disease was (3.37 ± 2.12) years. 17 cases had an AGA family history and 13 cases without. In group A, patients ranged in age from 21 to 49 years old, with an average age of (30.8 ± 7.58) years old; The duration of disease ranged from 0.5 to 8 years, with a mean course of (3.77 ± 2.38) years. In group B, patients ranged in age from 25 to 42 years old, with an average age of (30.4 ± 4.88) years old; The course of the disease was 0.3–6 years, with an average course of (3.02 ± 1.74) years (Table 1). Baseline data was comparable with no significant differences between the two groups. All enrolled patients were provided with written informed consent.

**TABLE 1:** Demographic data of subjects at baseline (SD, standard deviation).

	PRP with topical 5% minoxidil (A)	PRP with topical placebo (B)
**Age (years)**		
Mean ± SD	30.8 ± 7.58	30.4 ± 4.88
Range	21–49	25–42
**Duration of hair loss**		
Mean ± SD	3.77 ± 2.38	3.02 ± 1.74
Range	0.5–8	0.3–6
**AGA type (BASP)**		
M0	1	2
M1	6	4
M2	4	6
M3	3	3
F1	4	3
F2	3	7
F3	2	1
V1	10	10
V2	1	3
V3	0	1

### 2.2 Inclusion and exclusion criteria

#### 2.2.1 Inclusion criteria

1. Male patients with a clinical diagnosis of mild/moderate AGA and the diagnosis of AGA as the only cause of the patient's hair loss.
2. Patients over 18 years old signed informed consent.

#### 2.2.2 Exclusion criteria

1. History of use of minoxidil, finasteride, spironolactone, or glucocorticoids and immunosuppressant drugs for at least 3 months.
2. Patients with abnormal platelet count, coagulation disorders, or using anticoagulants.
3. Patients suffering from infectious diseases such as hepatitis, syphilis, and HIV or unable to tolerate automated methods for PRP preparation.
4. Local rupture or infection within the injection area.

### 2.3 Intervention and treatment protocol

Minoxidil and placebo were blinded by SAS9.4 software. Patients and researchers were blinded to the treatments. All patients were randomized by assigning blinded drugs and then allocated into two treatment arms:

*Group A (PRP+M)*: PRP prepared by automatic blood cell separator combined with topical 5% minoxidil therapy.

*Group B (PRP+P)*: PRP prepared by automatic blood cell separator combined with topical placebo therapy.

All patients received subcutaneous injections of PRP treatment (three times injections, at monthly intervals), combined with blind drugs use topically (twice a day for 3 months). Group A received treatment with topical 5%minoxidil (Shanxi Zhendong Ante Biopharmaceutical Co., LTD. [60 ml, 3g]), and group B was treated with a placebo topically.

#### 2.3.1 Automated method of PRP preparation

After the evaluation of patients’ blood vessels and build‐up of venous access, the automated blood cell separator (Fresenius COM.TEC, Germany) and the Plt‐5d (Platelet collection) procedure were used to prepare 30 ml of PRP by professionals of the transfusion department. PRP was divided into three sterile tubes (NEST 15 ml centrifuge tube) on average, frozen and stored at ‐40°C. PRP samples prepared by automated blood cell separator (automated method) were analyzed by automatic blood cell analyzer (BC‐7500). Platelet count in PRP was determined and compared with the whole blood samples of each patient (Table 2).

**TABLE 2:** Platelet concentration in whole blood and PRP (prepared by automated method).

Patient no.	Study group	Age (Y)	Blood platelet count	PRP platelet count
**S01**	B	42	160	547
**S02**	A	32	179	869
**S03**	A	25	234	568
**S04**	B	25	332	669
**S05**	B	29	327	886
**S06**	A	30	124	498
**S07**	B	32	249	1170
**S08**	A	41	227	496
**S09**	A	29	268	952
**S10**	A	27	338	886
**S11**	B	27	200	1015
**S12**	B	35	184	705
**S13**	A	35	162	877
**S14**	B	34	274	1020
**S15**	B	36	152	760
**S16**	A	32	292	1711
**S17**	A	24	208	708
**S18**	B	30	218	1065
**S19**	A	21	229	1052
**S20**	B	27	181	864
**S21**	B	28	215	663
**S22**	A	33	261	991
**S23**	A	22	249	847
**S24**	B	25	245	936
**S25**	B	25	272	926
**S26**	A	25	237	829
**S27**	B	33	184	849
**S28**	A	37	198	780
**S29**	A	49	275	967
**S30**	B	28	330	987

#### 2.3.2 PRP injection therapies

One tube of PRP was thawed before each treatment and injected into the scalp as soon as possible. The scalp and the surrounding 2 cm of the alopecia area were disinfected with iodophor before injection. The injection of PRP was used in a 1‐ml syringe with a 0.3 × 13.0 mm needle. Subcutaneous injection at a density of 0.1ml/cm^2^ was performed evenly in the opposite direction of hair growth without anesthesia. The scalp was disinfected again after injection, applying ice was appropriate to stop bleeding, and patients were instructed to avoid exposure to sunlight and other local irritants to keep the injection area dry for 24 hours. PRP injections were given at 4‐week intervals, with three consecutive treatments.

#### 2.3.3 Topical therapies

The usage and dosage of topical drugs were the same in both groups. Topical application started the next day after injection. Keep scalp dried before application, apply 1ml from the center to the periphery of the alopecia area, spray twice a day, then massage the scalp for 3–5 min.

### 2.4 Evaluation and assessment

For each patient, trichoscopic images were captured and measured by dermoscopy (Beijing Dermat Technology Co., LTD.). Trichoscopy (20x lens) was used to analyze hair density/quantity and average hair diameter in a dermoscopic unit (1.28 cm^2^) under the same physiological marker in the area of hair loss. For patients without physiological markers, the intersection of the binaural connection and the anterior median line, or the hair rotation was marked by tattooing for subsequent evaluations. Photographs were also taken by specialists at certain profiles (at specific angles of 0°, 45°, and 90°) for each patient in the same position and under the same light. The clinical efficacy of hair images was evaluated by two dermatologists before and after treatment. Patient satisfaction and adverse reactions were recorded after treatment. All patients were evaluated at baseline and in the 4th week after the last injection.

#### 2.4.1 Clinical efficacy evaluation

Significant: alopecia symptom disappeared, with hair regeneration up to 70%–100%. The shape and appearance of regrowth hair are basically similar to that of normal hair. Effective: the symptoms of hair loss disappeared, with hair regeneration up to 30%–69%. Ineffective: Symptoms of hair loss persisted, or the hair regrowth rate was less than 30%. Total efficiency = (number of significant cases + number of effective cases)/total cases×100%. The evaluation of clinical efficacy was performed by two dermatologists independently in a blind state.

#### 2.4.2 Patient satisfaction and adverse reactions

A self‐satisfaction questionnaire was followed up in the 4th week after the last PRP injection treatment. Based on patients' subjective evaluation of pruritus, dandruff, oil secretion, hair growth, and texture improvement, patient satisfaction was rated into three levels: very satisfied, satisfied, or dissatisfied. Meanwhile, the duration and severity of adverse reactions (erythema, pain, bleeding, etc.) during the treatment were recorded.

### 2.5 Statistics

Statistical software SPSS23.0 was used for statistical analysis with mean ± standard deviation ($\bar{x}\pm s$). Comparison between the two groups was conducted by student *t*‐test. *p* < 0.05 indicates that the difference is statistically significant.

## 3 RESULTS

The clinical efficacy of the two groups was evaluated in the 4th week after the last PRP injection. According to the comparison of hair images, we detected that hair became thicker, and hair color deepened and increased hair coverage after PRP therapy. According to the photographs of three enrolled patients, it was found that the global appearance and hair quality were improved in visual assessment (Figure 1). In the comparison of clinical efficacy, 14 patients in group A were shown to have clinical improvement compared to 11 patients in group B. Total efficiency was 93.3% (14/15) in group A, which was higher than that of group B 73.3% (11/15) (Table 3).

FIGURE 1Global photographic assessment at baseline and 3 months. Patient 1 Group A (PRP+M): Baseline 3 months. Patient 2 Group B (PRP+P): Baseline 3 months. Patient 3 Group A (PRP+M): Baseline 3 months. Patient 4 Group B (PRP+P): Baseline 3 months.

**Figure:**
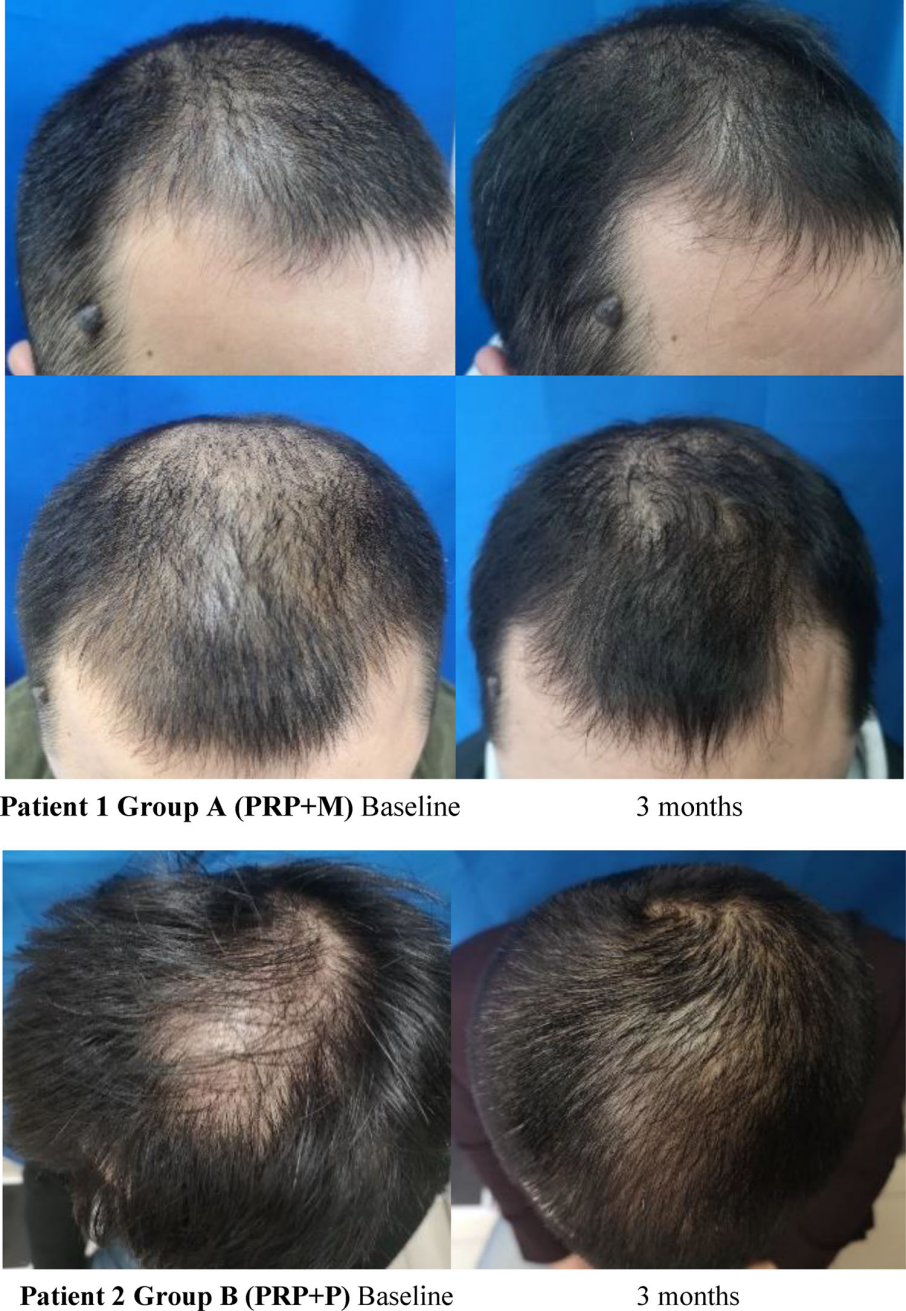

**Figure:**
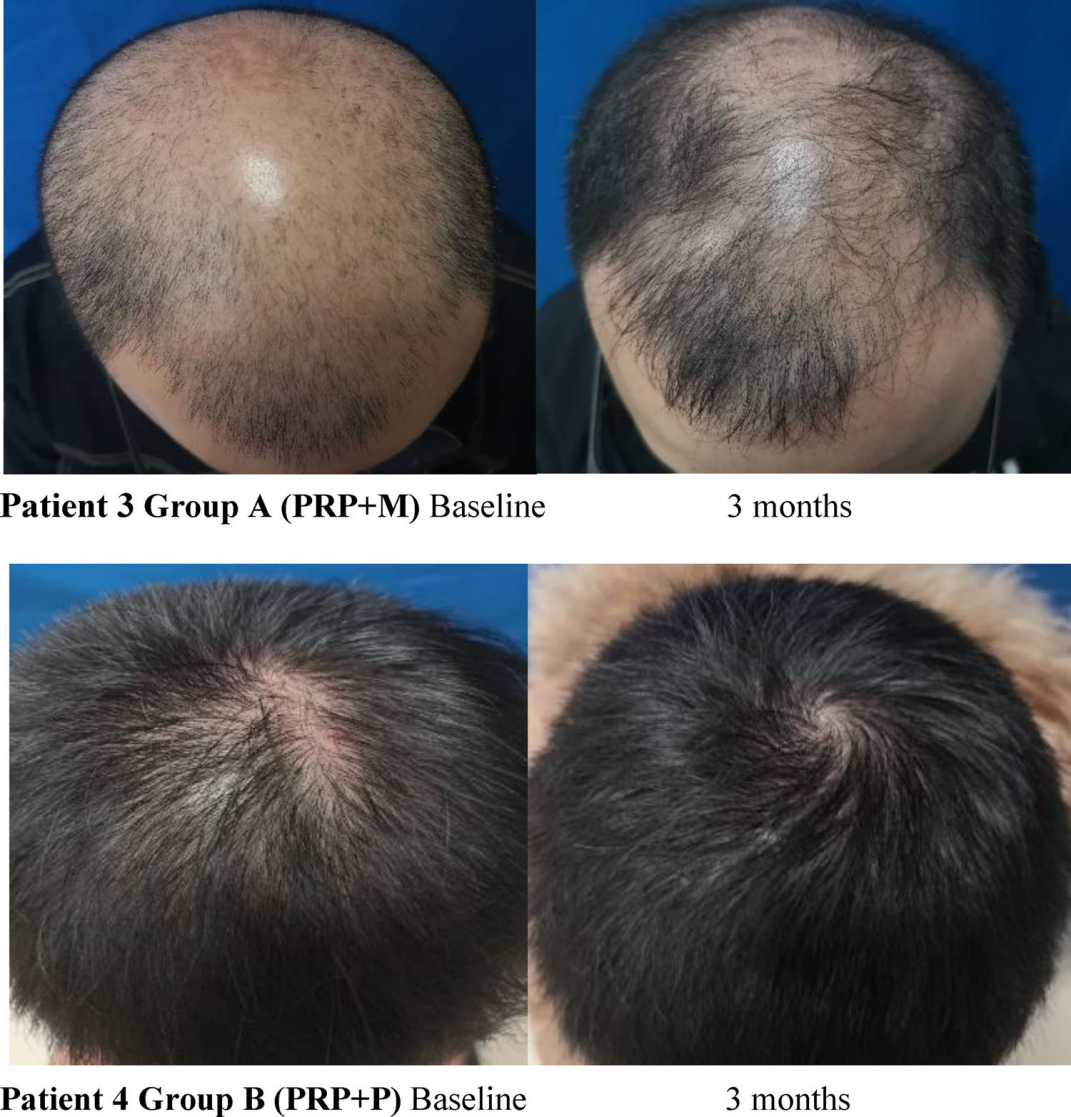

**TABLE 3:** Clinical efficacy.

	*N*	Significant effective	Effective	Ineffective	Total efficiency (%)
**Group A**	15	7	7	1	93.3
**Group B**	15	4	7	4	73.3

(χ^2^ = 2.618, *p* = 0.270)

**FIGURE 2:**
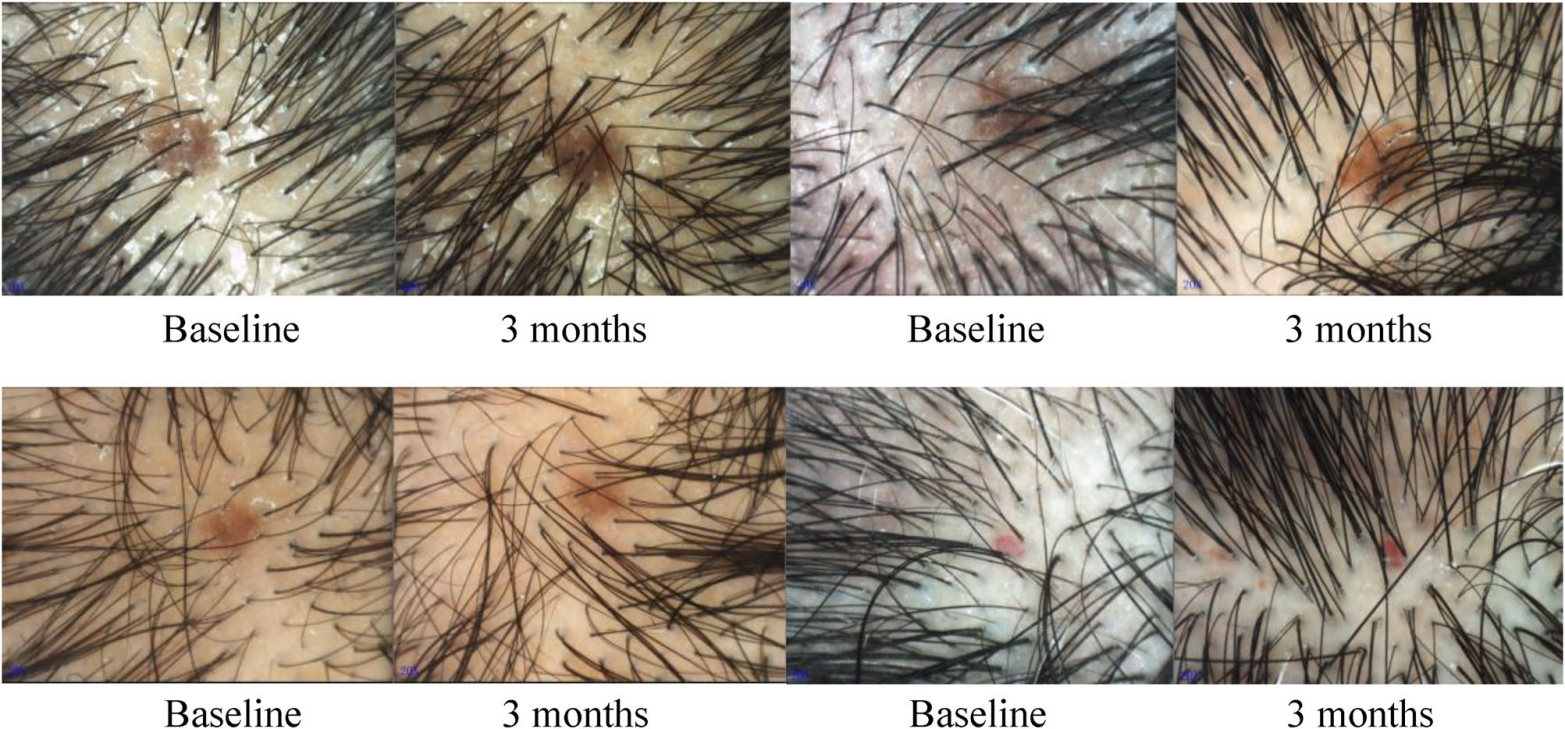
Trichoscopic comparative evaluation. Hair count, hair density, and mean hair diameter were assessed at baseline and 3 months. (Dermoscopy [polarized 20x])

**FIGURE 3:**
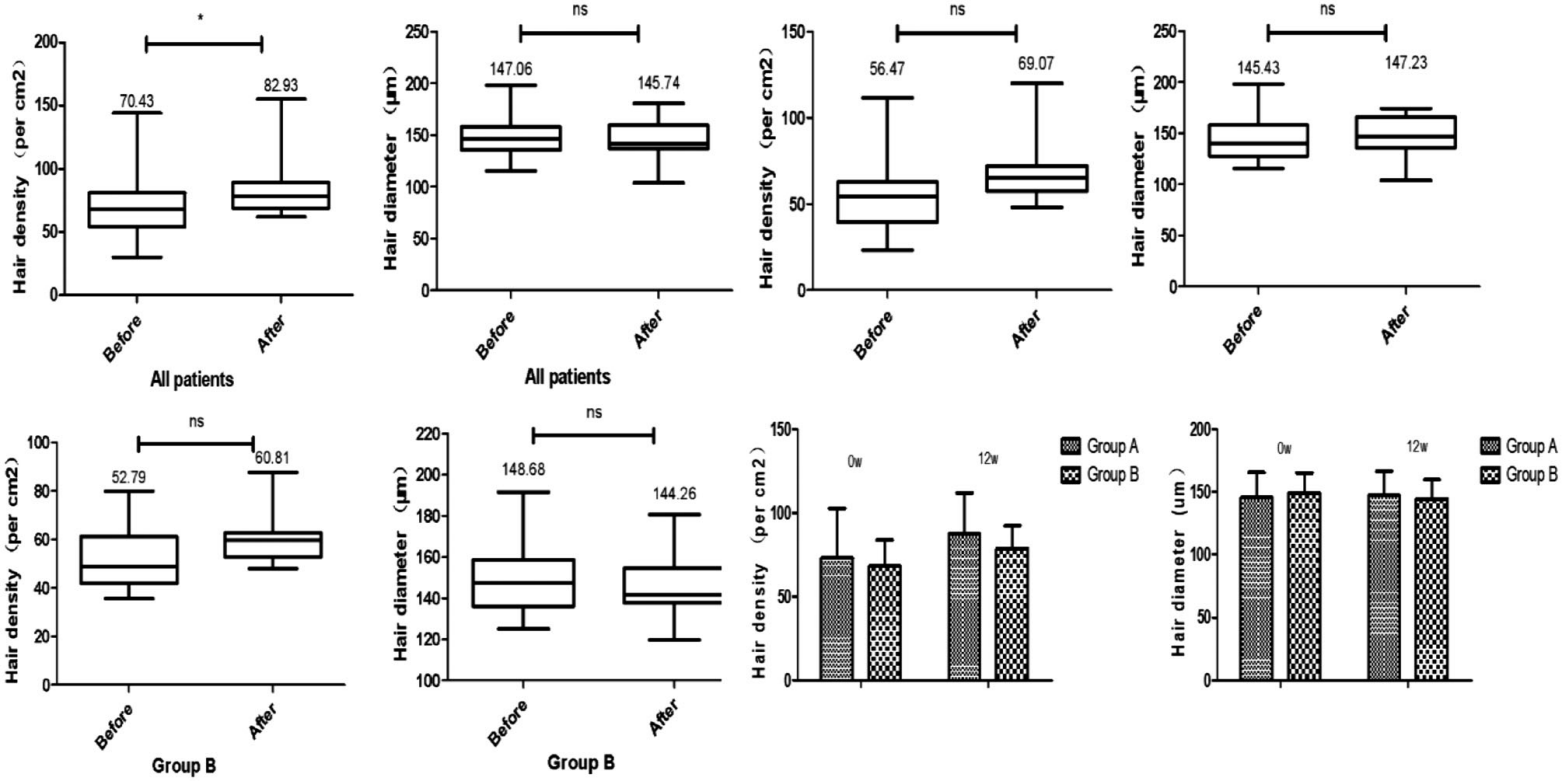
Comparison of average dermoscopic hair density and hair diameter before and after treatment.

On trichoscopic evaluation, increased hair diameter diversity (HDD) and positive brown peripilar sign around hair follicles were observed in almost 100% of the patients. Some patients had presentations like white and yellow dots, accompanied by increased scalp greasy and dandruff. In the comparison of dermoscopic (20x lens) hair analysis statistics, compared with baseline, the average hair density/quantity of all patients increased after treatment, which were 54.64 ± 18.32 versus 64.94 ± 16.07 and 70.43 ± 23.62 versus 82.93 ± 20.01, respectively. The difference was statistically significant (*p* < 0.05). While in comparison between groups, although the average hair density increased in group A was higher than that in group B, there was no significant difference (p = 0.26 > 0.05). Whereas no significant differences were detected in the mean hair diameter of both groups. After treatment, group A was 145.43 ± 20.13 versus147.23 ± 19.25 and group B was 148.68 ± 16.59 versus144.26 ± 15.39, respectively. There were no statistical differences in mean hair diameter between groups (*p* = 0.459 > 0.05). (Table 4, Figure 2, 3)

**TABLE 4:** Statistics of hair density, hair count, and mean hair diameter at baseline and 3 months.

	All patients		PRP + 5% Minoxidil (A)		PRP + Placebo (B)		Group A versus Group B
	Mean ± SD	*p*	Mean ± SD	*p*	Mean ± SD	*p*	*p*
**Hair density**							
Baseline	54.64 ± 18.32	0.024*	56.47 ± 23.15	0.119	52.79 ± 12.33	0.67	0.26
3 months	64.94 ± 16.07		69.07 ± 19.60		60.81 ± 10.71		
**Mean hair diameter (µm)**							
Baseline	147.06 ± 18.20	0.775	145.43 ± 20.13	0.805	148.68 ± 16.59	0.455	0.459
3 months	145.74 ± 17.19		147.23 ± 19.25		144.26 ± 15.39		
**Hair count (1.29 cm^2^)**							
Baseline	70.43 ± 23.62	0.031*	72.8 ± 29.85	0.152	68.07 ± 15.89	0.68	0.357
3 months	82.93 ± 20.01		87.47 ± 24.39		78.40 ± 13.79		

* *p* < 0.05

In terms of patient self‐satisfaction evaluation, five patients in group A were very satisfied, eight were satisfied and two were dissatisfied. The overall satisfaction was 86% (13/15). In group B, two patients were very satisfied, nine were satisfied and four were dissatisfied. The overall satisfaction was 73% (11/15). The overall satisfaction of group A was higher than group B, indicating that PRP combined with the topical 5% minoxidil group has higher satisfaction. The adverse reactions of PRP injection therapy were transient pain at the injection site and a burning sensation, which could be relieved after ice application and tolerated. Spot bleeding and erythema may subside spontaneously without a scar after injection. In the course of treatment, no serious adverse reactions occurred, except for one patient who experienced mild dizziness and nausea during injection, which was self‐alleviated after a few minutes. The primary outcome of our study was the effectiveness of the treatment demonstrated by physicians assessing the improvement in hair growth under trichography and photography. Secondary outcomes, as discussed above, were measured by patient self‐satisfaction and the occurrence of adverse reactions. The outcomes demonstrated the safety and effectiveness of the automated method prepared PRP injection combined with topical 5% minoxidil therapy.

## 4 DISCUSSION

AGA affects up to 50% of females and 80% of males.^9^ It starts after puberty and presents progressive development. Male pattern alopecia is based on the recession of the hairline, the top bald hair as a typical performance, part of the “horseshoe” appearance.^10^ AGA usually accompanies by scalp greasy, increased dandruff, pruritus, and other symptoms. AGA is a polygenic disease with complex inheritance, the exact etiology and pathogenesis remain unclear. Androgen as the main pathogenic factor of AGA, leads to gradual miniaturization of hair follicles, vellus hair‐like degeneration, and shortening hair period. Hair density is progressively reduced, causing hair thinning, hair follicle atrophy, and growth cessation thus specific area alopecia. This procedure eventually leads to the occurrence of AGA.^11^ AGA can be characterized by increased HDD under trichoscopy, peripilar sign/perifollicular pigmentation, and yellow dots.^12^

Minoxidil is currently the first‐line drug for the treatment of mild/moderate AGA. As an effective vasodilator, which is conducive to hair growth. The mechanism of this action may be related to various ways: potassium ion channels open, stimulate the proliferation and differentiation of hair follicle epithelial cells, promoting angiogenesis and vasodilation to increase local blood supply, directly accelerating the transformation of hair follicles from the resting phase to the anagen phase.^2^, ^13^ However, it is prone to relapse after drug withdrawal and requires long‐term maintenance, resulting in poor patient compliance.

PRP is a platelet‐concentrated plasma prepared by extracting autologous blood, with a higher concentration of platelets than blood. The regenerative potential of PRP is related to the levels of growth factors secreted and released by concentrated platelet α particles. PRP enriched with a variety of growth factors including platelet‐derived growth factor (PDGF), transforming growth factor (TGF), vascular endothelial growth factor (VEGF), epidermal growth factor (EGF), fibroblast growth factor (FGF), insulin‐like growth factor (IGF), and so forth. These growth factors affect the differentiation, proliferation, and survival of cells.^14^, ^15^ PDGF enriched in PRP is involved in the induction and maintenance of the anagen phase and plays a positive role in hair formation; IGF‐I has a significant effect on the growth rate of hair, prolonging the anagen phase and promoting the proliferation of hair follicle cells, thereby promoting hair growth.^16^, ^17^, ^18^, ^19^ Eppley et al.^20^ conducted a study to compare the concentrations of growth factors such as EGF, PDGF, and VEGF in PRP and whole blood. It was found that the concentrations of various growth factors in PRP were higher than those in whole blood samples, providing support for the positive effect of growth factors enriched in PRP on hair growth. The positive effectiveness and safety of PRP on hair were confirmed in a randomized, placebo‐controlled, half‐head study conducted by Gentile et al.,^21^ in which enrolled 23 AGA patients and received monthly injections. Hair regrowth was compared in patients treated with PRP and placebo after three monthly treatments. The results revealed that the patients presented clinical improvement in the mean number of hairs after treatment, without serious adverse reactions occurring. In addition, they also observed the number of epidermal Ki67+ keratinocytes and hair follicular bulge cells increased in histology, and the number of small blood vessels around hair follicles was also increased (*p* < 0.05), indicating that PRP could stimulate follicular and perifollicular angiogenesis. In a similar study, Anitua et al.^22^ treated 19 AGA patients with growth factor‐rich plasma for five injections, and achieved good clinical evaluation, showing increased hair density and diameter, improved vellus hair/terminal hair ratio, and histologically found increasing bulge stem cell niches and remodeling of dermo‐epidermal tissue. Ray et al.^23^ conducted a clinical observation study on topical minoxidil combined with PRP injection therapy, and randomly assigned 100 AGA patients to the topical minoxidil group and the minoxidil combined with the PRP injection group. After the treatment of 1 year, the combined treatment group was statistically superior to the monotherapy group in terms of photographic evaluation and hair diameter increase, which indicated that PRP combined with topical minoxidil had positive effects on hair growth.

The clinical efficacy of concentrated platelets depends mainly on the number of platelets and the concentration of growth factors.^24^ However, the amount of platelet enrichment and the number of granulocytes remaining in PRP varies from different PRP preparation methods, in which leukocytes and other pro‐inflammatory factors may promote the inflammatory response. Whereas inflammation is an important feature of AGA, higher inflammatory infiltration and reduced dermal vascularity can be observed in people with alopecia.^22^ In this study, PRP prepared by an automatic blood cell separator was determined to be 3–5 times higher than the concentration of whole blood platelets. The platelet enrichment of the automated method‐prepared PRP was high while the granulocyte content was low, so this kind of PRP with high purification quality may have better hair regeneration ability. In addition, the automated method prepared PRP has the advantages of one‐time automatic collection, convenient operation, high safety, stable quality, and long‐term storage under freezing. Besides, other blood components can be transfused back into the body during the procedure of PRP preparation, which can avoid blood waste.

In this study, all enrolled patients received injections of automated method‐prepared PRP. The results showed that the average hair density and hair counts of all patients after 3 months of treatment were significantly higher than that of pretreatment (*p* < 0.05). However, there was no significant difference in the average hair diameter before and after treatment. The reason for this result may be related to the analysis error of the tricholoscope, and the increase in the number of vellus hair after treatment thus causing the decrease in overall average hair diameter. PRP prepared by automatic blood cell separator therapy has certain efficacy in improving clinical symptoms of AGA. Patients who reported the promotion of hair texture, reduced hair loss, and scalp oil secretion after treatment, can achieve good patient satisfaction. This result is similar to the findings of Qu et al.,^25^ which confirmed the positive effect of PRP injection as an effective and reliable treatment for improving the clinical symptoms of AGA. The group of PRP prepared by an automated device with a combination of topical 5% minoxidil had higher patient satisfaction and better clinical efficacy under photographic evaluation, this treatment has a good application potential in the short term to improve the efficacy of AGA, which can be used as a novel clinical therapy for mild/moderate male androgenic alopecia without serious adverse reactions.

There are still some limitations in this study. On the one hand, due to the small sample size of this experiment, AGA patients of different severity have not been grouped and stratified compared, and for a short follow‐up period, the long‐term efficacy of PRP injection therapy could not be assessed. On the other hand, there is no unified standard for PRP preparation, concentration, injection method, course of treatment, and treatment interval of PRP, which may lead to bias in the efficacy evaluation. Therefore, further expansion of the sample size and longer follow‐ups are still needed to explore and determine the method to achieve optimal hair regrowth conditions.

In conclusion, our study provides further evidence that PRP injection is safe and effective for the treatment of mild/moderate AGA, and PRP prepared by automatic blood cell separators has high purification quality and high platelet concentration. The injection of automated method‐prepared PRP combined with topical 5% minoxidil therapy was superior to PRP monotherapy in terms of patient satisfaction and clinical efficacy for the treatment of mild/moderate male AGA, it is worthy of clinical promotion.

## CONFLICT OF INTEREST STATEMENT

The authors declare no conflicts of interest.

## ETHICS STATEMENT

The study was approved by the ethics committee of the Anhui Provincial Hospital (No. 2021‐ky195). All recruited subjects were aware of the study process and signed the written consent forms, agreed and cooperated with images taken, and supported the use of images and materials for academic research.

## Data Availability

None.
